# Cannabidiol, ∆^9^-tetrahydrocannabinol, and metabolites in human blood by volumetric absorptive microsampling and LC-MS/MS following controlled administration in epilepsy patients

**DOI:** 10.3389/fphar.2022.1038754

**Published:** 2022-10-24

**Authors:** Federica Pigliasco, Sara Malaca, Alfredo Fabrizio Lo Faro, Anastasio Tini, Giuliana Cangemi, Alessia Cafaro, Sebastiano Barco, Antonella Riva, Angelica Pisati, Elisabetta Amadori, Pasquale Striano, Adriano Tagliabracci, Marilyn Ann Huestis, Francesco Paolo Busardò

**Affiliations:** ^1^ Department of Neurosciences, Rehabilitation, Ophthalmology, Genetics, Maternal and Child Health (DINOGMI), University of Genoa, Genoa, Italy; ^2^ Department of Excellence-Biomedical Sciences and Public Health, Università Politecnica delle Marche, Ancona, Italy; ^3^ Chromatography and Mass Spectrometry Section, Central Laboratory of Analyses, IRCCS Istituto Giannina Gaslini, Genoa, Italy; ^4^ Department of Internal Medicine, Pharmacology & Toxicology Unit, University of Genoa, Genoa, Italy; ^5^ Paediatric Neurology and Muscular Disease Unit, IRCCS Istituto Giannina Gaslini, Genoa, Italy; ^6^ Institute of Emerging Health Professions, Thomas Jefferson University, Philadelphia, PA, United States

**Keywords:** cannabinoids, medical cannabis, serum, CBD metabolites, UHPLC-MS/MS

## Abstract

Cannabidiol (CBD) exhibits anti-inflammatory, anxiolytic, antiseizure, and neuroprotective proprieties without addictive or psychotropic side effects, as opposed to Δ^9^-tetrahydrocannabinol (THC). While recreational cannabis contains higher THC and lower CBD concentrations, medical cannabis contains THC and CBD in different ratios, along with minor phytocannabinoids, terpenes, flavonoids and other chemicals. A volumetric absorptive microsampling (VAMS) method combined with ultra-high-performance liquid chromatography coupled with mass spectrometry in tandem for quantification of CBD, THC and their respective metabolites: cannabidiol-7-oic acid (7-COOH-CBD); 7-hydroxy-cannabidiol (7-OH-CBD); 6-alpha-hydroxy-cannabidiol (6-α-OH-CBD); and 6-beta-hydroxycannabidiol (6-β-OH-CBD); 11- Hydroxy-Δ^9^-tetrahydrocannabinol (11-OH-THC) and 11-Nor-9-carboxy-Δ^9^-tetrahydrocannabinol (THCCOOH). After overnight enzymatic glucuronide hydrolysis at 37°C, samples underwent acidic along with basic liquid-liquid extraction with hexane: ethyl acetate (9:1, v/v). Chromatographic separation was carried out on a C18 column, with the mass spectrometer operated in multiple reaction monitoring mode and negative electrospray ionization. Seven patients with intractable epilepsy were dosed with various CBD-containing formulations and blood collected just before their daily morning administration. The method was validated following international guidelines in toxicology. Linear ranges were (ng/ml) 0.5–25 THC, 11-OH-THC, THCCOOH, 6-α-OH-CBD and 6-β-OH-CBD; 10–500 CBD and 7-OH-CBD; and 20–5000 7-COOH-CBD. 7-COOH-CBD was present in the highest concentrations, followed by 7-OH-CBD and CBD. This analytical method is useful for investigating CBD, THC and their major metabolites in epilepsy patients treated with CBD preparations employing a minimally invasive microsampling technique requiring only 30 µL blood.

## 1 Introduction

The most researched phytocannabinoids are cannabidiol (CBD) and Δ^9^-tetrahydrocannabinol (THC) (Degenhardt et al., 2017; Barco et al., 2018). CBD exhibits anti-inflammatory, antiseizure, anxiolytic, and neuroprotective proprieties without addictive or psychotropic effects, as opposed to THC (Pigliasco et al., 2020). While recreational cannabis generally contains high THC concentrations, medical cannabis contains THC and CBD in varying amounts, along with minor terpenes, flavonoids, phytocannabinoids, and other chemicals (Malaca et al., 2021).

There is growing interest in cannabis-based therapies and clinical applications (Lattanzi et al., 2019, 2020; Arzimanoglou et al., 2020). Different CBD products are available on the market with effects varying based on purity, formulation, and concentration. The European Medicines Agency has authorized Epidyolex^®^, a pure CBD oral solution. As an add-on therapy for drug-resistant epilepsies including Dravet syndrome, tuberous sclerosis complex and Lennox-Gastaut syndrome (Dubois et al., 2020).

Therapeutic drug monitoring (TDM) of a variety of antiseizure medications (ASMs) is critical in therapeutic management of patients with epilepsy. TDM is particularly useful in emerging clinical practice for cannabis-based therapies to identify the dose associated with an optimal response (Patsalos et al., 2018; Striano et al., 2008; Brandt, 2019). Although TDM is often performed on plasma or serum samples, a major challenge is a need for repeated venipunctures, which is stressful, especially for children. Dried blood spots and other microsampling techniques offer advantages including easy, rapid, and less invasive sample collection, low sample volumes of 10–50 μL, minimal sample preparation and safe sample handling with minimum risk of transmission of infectious diseases (Biagini et al., 2020).

Other analytical methods focused on CBD and metabolite identification and quantification in different biological matrices, but none included simultaneous quantification with the volumetric absorptive microsampling (VAMS) method (Barco et al., 2018; Dubois et al., 2020; Pérez-Acevedo et al., 2020, 2021; Pichini et al., 2020, 2021; Pigliasco et al., 2020; Busardò et al., 2021; Malaca et al., 2021). This method quantifies CBD, THC, cannabidiol-7-oic acid (7-COOH-CBD); 7-hydroxy-cannabidiol (7-OH-CBD); 6-alpha-hydroxy-cannabidiol (6-α-OH-CBD); and 6-beta-hydroxycannabidiol (6-β-OH-CBD); 11-Hydroxy-Δ^9^-tetrahydrocannabinol (11-OH-THC) and 11-Nor-9-carboxy-Δ^9^-tetrahydrocannabinol (THCCOOH) in whole blood collected with VAMS and analyzed with ultra-high performance liquid chromatography coupled with mass spectrometry in tandem (UHPLC-MS/MS). In our previous research, we demonstrated that CBD plasma concentrations were comparable to those measured in venous or capillary blood with VAMS, allowing the use of this microsampling fingerpick device (Barco et al., 2017; D’Urso et al., 2019; Pigliasco et al., 2020). The validated method quantified cannabinoids in children’s blood with drug-resistant epilepsy.

## 2 Materials and methods

### 2.1 Chemicals and reagents

Standards for CBD, 7-OH-CBD, 7-COOH-CBD, 6-α–OH–CBD and 6-β–OH–CBD, THC, 11-OH-THC, and THCCOOH were obtained from Dalton Research Molecules (Toronto, ON, Canada) and deuterated internal standards (ISTD) THC-d_3_, 11-OH-THC-d_3_, THCCOOH-d_3_ and CBD-d_3_ were purchased from Cayman Chemical (Ann Arbor, MI, United States) and stored at −20°C until use. LC-MS grade water, LC grade acetone, formic acid and acetonitrile acquired from Sigma-Aldrich^®^ (Milano, Italy). Ammonium formate 5 mM was prepared with 97% pure ammonium formate ammonium salt (Sigma-Aldrich^®^) dissolved in LC-MS grade water. Beta-glucuronidase from *E. Coli* (>20.000 units mg/protein) was obtained from Sigma Aldrich^®^ (Milano, Italy).

### 2.2 Instrumental conditions for UHPLC-MS/MS

A Waters^®^ Xevo^®^ TQ-S micro mass spectrometer (triple quadrupole), equipped with an electrospray ionization source operating in both negative and positive-ion mode (ESI), was used to conduct the UHPLC-MS/MS analysis. The instrument was interfaced with an ACQUITY UPLC^®^ I-Class (Waters^®^; Milan, Italy). Data were collected using the MassLynx^®^ program version 4.1 (Waters^®^, Milano, Italy). A Waters^®^ ACQUITYTM PREMIER UPLC^®^ BEH C18 column (100 × 2.1 mm, 1.7 μm) was used for separation. Run time was 17 min with mobile phases (A) water with ammonium formate 5 mM pH 7.5 and (B) acetonitrile at a flow rate of 0.4 ml/min. The gradient program went from 5% B for 0.25 min to 30% B after 1 min, 80% B after 11.5 min and held for 0.5 min, 100% B after 11.55 min till 13.5 min, and then back to 5% B after 13.55 min and held for the remaining 17:00 min. Column oven and autosampler temperatures were 50°C and 10°C, respectively. The mass spectrometer was operated in multiple reaction monitoring (MRM) mode, with two transitions for each analyte and ISTD (see Table 1). By individually injecting neat standards into methanol and ramping cone voltage and collision energy, MS parameter settings were made to be fully optimized. (see Table 1). The ESI conditions were optimized to source temperature 150°C, capillary voltage −2.8 kV, cone gas flow rate 0.18 ml/min, desolvation temperature 650°C, and desolvation gas flow rate 1200 L/h. The scan speed (dwell time) was 0.023 s.

**TABLE 1:** Mass spectrometry parameters for analytes and internal standards.

Compounds	Internal Standard	Cone voltage (eV)	Q1 mass (m/z)	Quantification transition		Confirmation transition		RT (min)
				Q3 mass (m/z)	CE (eV)	Q3 mass (m/z)	CE (eV)	
Standards								
7-COOH-CBD	11-OH-THC-d_3_	40	343.1	179.2	20	231.2	26	5.57
6-α–OH–CBD	11-OH-THC-d_3_	30	329.2	158.2	32	173.1	28	6.17
7-OH-CBD	11-OH-THC-d_3_	25	329.1	261.2	20	268.1	24	6.42
6-β–OH–CBD	11-OH-THC-d_3_	30	329.2	158.2	26	173.2	34	6.50
THCCOOH	THCCOOH-d3	40	345.1	193.1	24	299.2	24	7.61
11-OH-THC	11-OH-THC-d3	30	331.2	193.1	24	201.1	24	8.23
CBD	CBD-d_3_	45	315.2	123	34	189.1	22	9.79
THC	THC-d3	45	315.2	123	34	193.1	22	11.27
Internal Standards								
THCCOOH-d_3_	-	40	348.1	196.2	26	-	-	7.61
11-OH-THC-d_3_	-	30	334.2	196.1	30	-	-	8.22
CBD-d_3_	-	45	316.1	110.1	45	248.2	45	9.77
THC-d_3_	-	45	318.2	123	34	196.1	22	11.27

CBD, Cannabidiol; 7-COOH-CBD, 7-Carboxy-cannabidiol; 7-OH-CBD, 7-Hydroxy-cannabidiol; 6-α–OH–CBD, 6-α-Hydroxy-cannabidiol; 6-β–OH–CBD, 6-β-Hydroxy-cannabidiol; THC, Δ^9^-Tetrahydrocannabinol; 11-OH-THC, 11-Hydroxy-Δ^9^-tetrahydrocannabinol; THCCOOH, 11-Nor-9-carboxy-Δ^9^-tetrahydrocannabinol; CE, collision energy; RE, retention time.

### 2.3 Preparation of quality control samples and calibration standards

Standard stock solutions with all five non-deuterated standards were prepared in methanol at 1 mg/ml, 100 μg/ml, 10 μg/ml, and 1 μg/ml. ISTD stock solution with THC-d_3_, THCCOOH-d_3_, 11-OH-THC-d_3_ and CBD-d_3_ was prepared in methanol at 1 μg/ml. Due to the unavailability of deuterated standards for the analytes, the deuterated 11-OH-THC standard was used for the CBD metabolites. Glass vials containing the stock solutions were kept at −20°C.

Pre-screened blood samples were donated by Department of Neurosciences, Rehabilitation, Ophthalmology, Genetics, Maternal and Child Health (DI-NOGMI, University of Genoa, Genoa, Italy) as material discarded during current clinical practice. During method validation, samples were evaluated to rule out any potential sources of chromatographic interferences and then mixed to form a homogeneous pool of blank samples for the preparation of calibration standards and quality control samples.

Calibrator working solutions were prepared by diluting standard stock solutions with methanol (0.25, 0.5, 1, 2, 5 and 25 ng/ml for THC, 11-OH-THC, THCCOOH, 6-α–OH–CBD and 6-β–OH–CBD; 1.5, 25, 50, 100, 250 and 500 ng/ml for CBD and 7-OH-CBD; 3.5, 20, 100, 500, 1000 and 5000 ng/ml for 7-COOH-CBD). Low, medium and high-quality control (QC) working solutions were prepared daily from the standard stock solutions in methanol. THC, 11-OH-THC, THCCOOH, 6-α–OH–CBD and 6-β–OH–CBD QC concentrations were 0.75, 1.5 and 20 ng/ml, CBD and 7-OH-CBD QC concentrations were 5, 80 and 400 ng/ml and 7-COOH-CBD QC concentrations were 10, 350 and 4000 ng/ml.

### 2.4 Sample preparation

Since prior research and our preliminary experiments in real samples revealed that CBD metabolites were present as glucuronides, blood samples were extracted following enzymatic hydrolysis (Bergamaschi et al., 2013; Ujváry and Hanuš, 2016). Glucuronide hydrolysis was carried out by adding 2 μL ISTD solution (100 ng/ml), beta-glucuronidase (*E. Coli* >20,000 units mg/protein) was added to 30 ml of blood in a VAMS tip, followed by dilution in 800 ml of water and heated overnight at 37°C. After hydrolysis, acidic and basic extractions were performed to extract all metabolites based on their acid-base properties. For the basic extraction, 100 μL ammonium hydroxide (pH 9) was added to the hydrolyzed sample after the addition of 4 ml hexane: ethyl acetate (9:1). Samples underwent horizontal agitation for 30 min. Samples were centrifuged for 3 min at 3500 rpm, and the supernatant was then transferred to a clean tube. The samples were centrifuged for 3 minutes at 3500 rpm and the supernatant was then transferred to a clean tube. The remaining aqueous phase was then treated with 15 μL of formic acid (≥99.9%) (pH 3) for acid extraction. Four mL of hexane: ethyl acetate (9: 1) was added to the sample. The tubes were then capped, vortexed for 10 s, mixed for 10 min and centrifuged at 5000 g for 5 min . Both extractions’ supernatants were combined in a glass tube (Safe-Lock Tube^®^, Eppendorf, Milano, Italy) and dried under nitrogen. Samples were resuspended with 100 µL water: methanol (1:1), transferred into autosampler glass vials, and 10 μL injected onto the chromatographic system.

### 2.5 Validation of the analytical method

#### 2.5.1 Sensitivity

Sensitivity was determined by analyzing four replicates of negative samples and examination of the signal/noise ratio. The standard deviation (SD) of the mean noise level over the retention time window of analytes was used to determine the detection limit (LOD = 3 SD) and the quantification limit (LLOQ = 10 SD). The calculated LLOQ had to demonstrate precision and accuracy within the 20% relative SD and relative error, respectively, in order to be acceptable.

#### 2.5.2 Selectivity and carryover

Selectivity evaluated the capacity of analytes to be identified in the presence of matrix elements. Blank blood samples were assayed to identify possible endogenous interferences. In addition, blank blood samples were assayed for possible exogenous drug interferences. For this, other commonly encountered analytes (such as common drugs or metabolites) encountered in routine work were analyzed with fortified matrix samples at high therapeutic or lethal concentrations. The acceptance criteria were no signal/noise ratio higher than 3 at ± 0.2 min of the retention time of the analytes (ranging from 5.57 to 11.27 min) in the quantitative and qualitative ions. To test for carryover, blank blood samples were analyzed immediately after the highest calibrator. Carryover was the highest fortified concentration at which no analyte carryover above the method’s LOD was observed in the blank matrix.

#### 2.5.3 Calibration curve

Six calibrators were assayed on five separate days to establish the calibration curve. The peak area ratio of each compound and its corresponding ISTD were plotted against each analyte’s concentration. The minimally acceptable linearity requires a coefficient of correlation (*r* ^2^) ≥ 0.99 and each calibrator quantifying within ±20% of target concentration. Dilution integrity was checked for over-the-curve samples with concentrations 10 and 50 times higher than the highest calibrators, verifying precision and accuracy to be within 15%.

#### 2.5.4 Imprecision and bias

Imprecision was expressed as the RSD (%), and bias was calculated as (determined concentration)/(nominal concentration)×100%. Acceptance criteria for intra- and inter-assay imprecision were CV ≤ 20% and bias ≤15%. To evaluate intra-assay imprecision, six blank blood samples each were fortified with the target analytes at three different concentrations (low, medium, and high QC) and analyzed on the same day. Evaluation of inter-assay imprecision and bias were performed over 5 days with a minimum of six concentrations.

#### 2.5.5 Matrix effect and recovery

Matrix effect was determined by comparing peak areas of the extracted blank samples fortified with standards after the extraction procedure with the peak areas of pure diluted substances. Recovery was determined by comparing peak area of the extracted compounds fortified before extraction to the peak area obtained from samples fortified post-extraction (representing 100% recovery). The ISTD mixture was added to samples after extraction.

#### 2.5.6 Stability

Compounds’ stability in blood was evaluated through repeated analysis (n = 5) of QC samples after three freeze-thaw cycles (storage at −20°C) on the compounds stability in blood was evaluated by repeated analysis (n = 5) of QC samples. In addition, short term (24 and 48 h) and mid-term (1 month) stability were assessed using five different aliquots of QC stored at −20°C. The stability was expressed as a percentage of the initial concentration (first analyzed batch) of the analytes in QC.

### 2.6 Application to patients’ samples

The analytical method’s applicability was demonstrated using real clinical samples from patients taking different CBD formulations. (Epidyolex^®^, CBD oil, CBD oil Enecta and CBD crystal) for the treatment of drug-resistant epilepsy of different etiologies (see Table 2). Blood samples were collected in the morning before daily dose administration using the 30 µL VAMS devices (MITRA^®^, Neoteryx, 105 Torrance, CA, United States) for capillary blood collection. Capillary VAMS were obtained in accordance with the manufacturer’s recommendations: before pricking the patient’s finger with a micro-needle, the area was disinfected and after the first drop of blood was removed, the VAMS tip was placed in contact with the surface of the second drop to absorb the matrix. The study was approved by the Regional Ethical Committee (CER Liguria: 056/057/058/059-2019) and written informed consent was signed by patients or caregivers.

**TABLE 2:** Patients’ demographic, clinical, and treatment data.

Patient ID	Age (y), gender	Weight (Kg)	Epilepsy disorder	CBD formulation, dose (mg/Kg/day), treatment duration (d)	Concomitant drugs
1	9, ♂	24	Dravet	Epidyolex oral solution, 20	STP, VPA
			Syndrome	820	
2	8, ♀	23.6	Aicardi	Galenic CBD oil, 3.9, 1125	LTG
			Syndrome		
3	15, ♀	29	Noonan	Galenic CBD oil 24%, 7.2, 240	VPA, LCM, LZP
			Syndrome		
4	12, ♂	28.4	Focal non-lesional	CBD crystals & Bedrolite, 26.4 and 10.6, 2130	LEV, CLB
			epilepsy		
5	20, ♀	40	Focal non-lesional	CBD crystals, 7.5, 1030	VPA, FBM, NTZ
			epilepsy		
6	3, ♂	11	Infantile spasms/West Syndrome	Epidyolex oral solution, 16.3, 30	VPA, PB, CLB
7	8, ♂	35	Focal non-lesional	Galenic CBD oil, 10.8, 850	PB
			epilepsy		

CLB, clobazam; FBM, felbamate; LEV, levetiracetam; LCM, lacosamide; LTG, lamotrigine; LZP, lorazepam; NTZ, nitrazepam; PB, phenobarbital; STP, stiripentol; VPA, valproate.

## 3 Results

**FIGURE 1:**
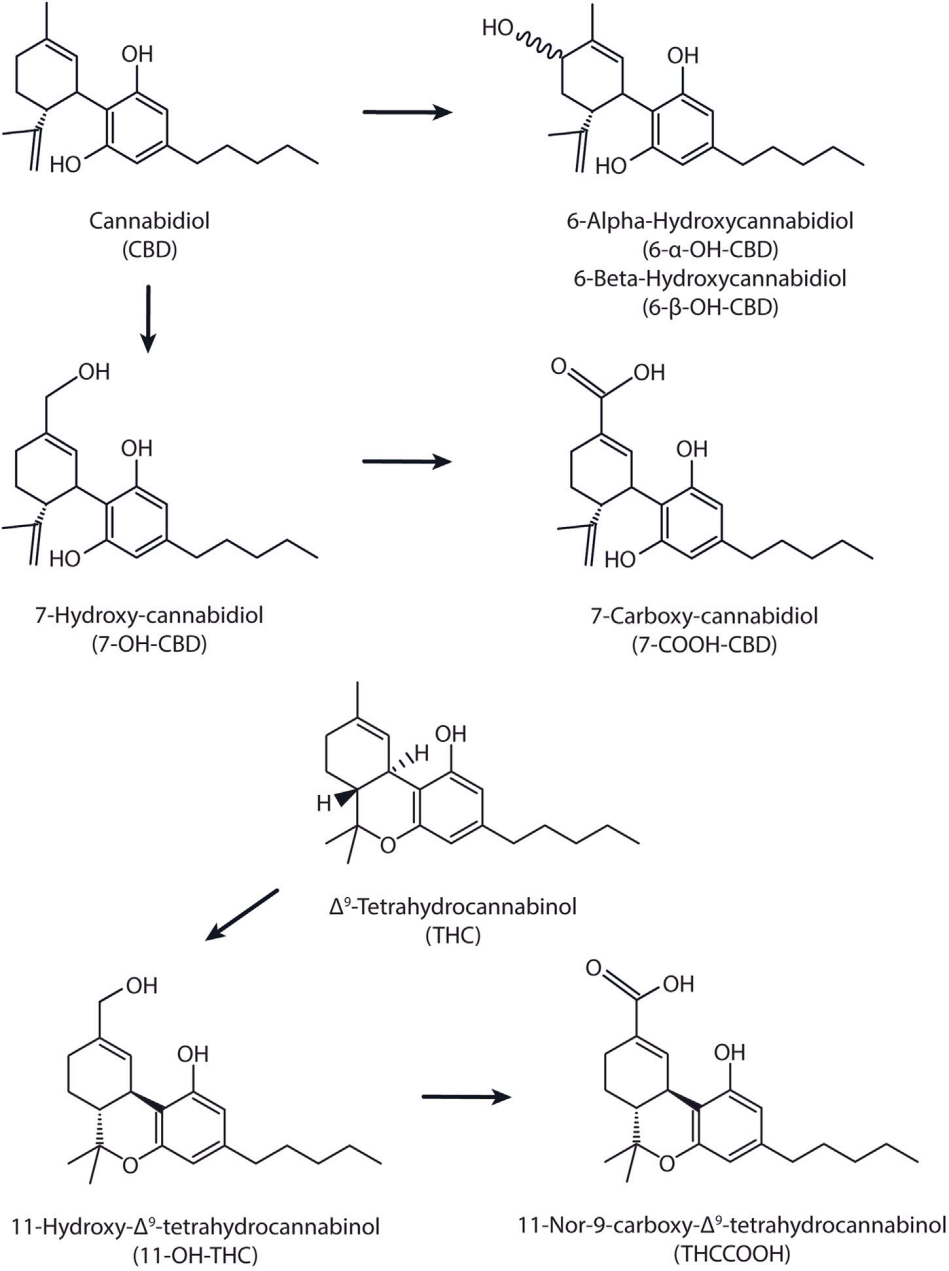
Chemical structures for CBD, THC, and their respective metabolites.

Previous analytical methods determined CBD, THC and metabolites (Figure 1) by UHPLC-MS/MS methods with VAMS collection (Dubois et al., 2020; Pigliasco et al., 2020) but no assay is currently available to simultaneously quantify all of these analytes in whole blood.

### 3.1 Validation of the analytical method

The method was validated over 5 days in blood samples following the most recent criteria for bioanalytical method development and validation (Peters et al., 2018; Wille et al., 2018), Linearity, sensitivity [limits of detection (LOD) and quantification (LOQ)], selectivity, accuracy, imprecision and carryover were calculated using five daily replicates of calibrators (six for each calibration curve) and five replicates of the three QC samples. Method validation results, presented in Tables 3, 4 were following the internationally established criteria (Peters et al., 2018; Wille et al., 2018). No relevant degradation was observed after any of the three freeze/thaw cycles, with differences in the initial concentration less than 15% for all the compounds under investigation. Similar results (differences from the initial concentration always lower than 15%) were obtained for the case of short-term and mid-term stability tests, confirming the validity of stored samples for analysis.

**TABLE 3:** Linearity, limits of detection (LOD) and limits of quantification (LOQ) for analytes under investigation in blood samples.

Analytes	Determination coefficient *r* ^2^	LOD ng/mL	LOQ ng/mL
CBD	0.996 ± 0.003	0.50	1.50
7-OH-CBD	0.999 ± 0.002	0.50	1.50
7-COOH-CBD	0.999 ± 0.002	1.10	3.50
6-α–OH–CBD	0.996 ± 0.004	0.10	0.25
6-β–OH–CBD	0.994 ± 0.003	0.10	0.25
THC	0.997 ± 0.002	0.10	0.25
11-OH-THC	0.998 ± 0.002	0.10	0.25
THCCOOH	0.998 ± 0.001	0.10	0.25

CBD, Cannabidiol; 7-COOH-CBD, 7-Carboxy-cannabidiol; 7-OH-CBD, 7-Hydroxy-cannabidiol; 6-α–OH–CBD, 6-α-Hydroxy-cannabidiol; 6-β–OH–CBD, 6-β-Hydroxy-cannabidiol; THC, Δ^9^-Tetrahydrocannabinol; 11-OH-THC, 11- Hydroxy-Δ^9^-tetrahydrocannabinol; THCCOOH, 11-Nor-9-carboxy-Δ^9^-tetrahydrocannabinol; LOD, limit of detection; LOQ, limit of quantification.

**TABLE 4:** Validation parameters for cannabinoid analytes under investigation in blood samples.

Analytes	Intra-assay accuracy %CV			Inter-assay accuracy %CV			Intra-assay imprecision %CV			Inter-assay imprecision %CV			Recovery (%)		
	L	M	H	L	M	H	L	M	H	L	M	H	L	M	H
7-COOH-CBD	5.1	2.4	5.7	7.9	2.5	4.1	6.9	2.4	4.7	7.0	2.6	4.8	95.7	95.9	97.9
6-α–OH–CBD	7.9	9.1	2.8	7.8	8.4	3.3	3.1	3.5	2.9	7.9	3.0	3.7	96.5	84.3	78.2
7-OH-CBD	7.8	7.5	5.2	6.9	7.7	4.7	8.8	4.5	3.6	6.8	3.1	2.9	93.7	92.2	98.1
6-β–OH–CBD	7.9	5.8	7.8	9.6	8.6	5.2	3.9	2.8	1.5	9.4	6.2	6.5	81.7	87.9	89.9
THC-COOH	4.2	8.3	5.0	58	8.6	4.8	5.8	3.9	2.2	5.8	5.8	6.5	83.5	94.0	93.2
11-OH-THC	9.9	10.4	9.1	9.5	8.5	5.5	2.9	3.7	2.7	4.2	3.8	7.0	77.8	86.4	86.4
CBD	10.2	4.1	2.3	7.8	5.8	3.8	9.2	4.8	1.1	6.8	6.4	5.1	69.0	63.2	64.5
THC	7.1	4.9	7.0	6.9	7.4	6.9	5.8	4.0	2.6	6.1	6.7	2.8	80.1	85.6	88.9

CBD, Cannabidiol; 7-COOH-CBD, 7-Carboxy-cannabidiol; 7-OH-CBD, 7-Hydroxy-cannabidiol; 6-α–OH–CBD, 6-α-Hydroxy-cannabidiol; 6-β–OH–CBD, 6-β-Hydroxy-cannabidiol; THC, Δ^9^-Tetrahydrocannabinol; 11-OH-THC, 11-Hydroxy-Δ^9^-tetrahydrocannabinol; THCCOOH, 11-Nor-9-carboxy-Δ^9^-tetrahydrocannabinol; CV, coefficient of variation; L, low; M, medium; H, high.

### 3.2 Analysis of patients’ samples

For proof of concept, the VAMS collection and analytical method were applied to seven samples from seven patients receiving various CBD formulations each at different therapeutic dosages. Four males (ages 3–12 years; weight: 11–28.4 kg) and three females (age range: 8–20 years; weight: 23.6–40 kg) were treated at the Giannina Gaslini Children’s Hospital provided samples. The study was approved by the Regional Ethical Committee (CER Liguria: 056/057/058/059-2019) and written informed consent was provided by patients or caregivers. Table 5 summarizes the patients’ results.

**TABLE 5:** Formulations, doses, and concentrations of CBD, THC, and their respective metabolites in patients’ blood samples collected with the VAMS technique after CBD therapy.

ID	Formulation	CBD mg/kg/day	Concentration ng/mL							
			6-α-OH-CBD	6-β-OH-CBD	7-OH-CBD	CBD-COOH	CBD	11-OH-THC	THC-COOH	THC
1	Epidyolex oral solution	20	16.8	17.4	399	7335	109	N.D.	N.D.	N.D.
2	Galenic CBD Oil	3.9	28.9	38.8	883	6677	2503	N.D.	N.D.	N.D.
3	CBD Oil 24%	3.9	0.9	1.0	53.5	357	19.1	N.D.	N.D.	N.D.
4	CBD crystal & Bedrolite	250	8.2	5.8	498	15,371	333	N.D.	N.D.	N.D.
5	CBD crystal	100	2.0	4.0	77.3	211	39.0	N.D.	N.D.	N.D.
6	Epidyolex oral solution	0.9	2.6	6.2	266	1120	88.7	N.D.	N.D.	N.D.
7	Galenic CBD oil	18 drops	1.9	1.9	59.4	423	1878	N.D.	N.D.	N.D.

CBD, Cannabidiol; 7-COOH-CBD, 7-Carboxy-cannabidiol; 7-OH-CBD, 7-Hydroxycannabidiol; 6-α–OH–CBD, 6-α-Hydroxycannabidiol; 6-β–OH–CBD, 6-β-Hydroxycannabidiol; 11-OH-THC, 11-Hydroxy- Δ^9^-tetrahydrocannabinol; THCCOOH, 11-Nor-9-carboxy-Δ^9^-tetrahydrocannabinol THC, Δ^9^-Tetrahydrocannabinol; N.D., not detected.

## 4 Discussion

An analytical method was validated for the determination of CBD, THC, and their respective metabolites and later applied to clinical samples. Blood samples were collected from seven patients under treatment with CBD formulations. Blood CBD concentrations were higher for the patients treated with Galenic CBD oil (patients #2 and #7) compared to patients treated with other CBD preparations. 7-COOH-CBD, the inactive metabolite, was present in the highest concentrations, followed by 7-OH-CBD, and CBD. 6-α–OH–CBD and 6-β–OH–CBD concentrations were always lower than concentrations of the other CBD metabolites, but for the first time were detected in all patients’ samples, with the highest concentrations in patient #2. In previous studies, these two analytes were undetectable in some patients’ samples [4]. THC and its metabolites, THCCOOH and 11-OH-THC, were not detected, indicating that the CBD formulations contained little if any THC.

In conclusion, this simple and rapid UHPLC-MS/MS method enabled robust and sensitive quantification of CBD, THC, and their respective metabolites, with good precision, accuracy, and efficiency. Also, blood sample collection by a microsampling technique (VAMS) is a major advantage when dealing with patients, especially children to avoid invasive procedures during repeated venipunctures.

## Data Availability

The original contributions presented in the study are included in the article/supplementary material, further inquiries can be directed to the corresponding author.
